# From the most to the least flexible nutritional profile: Classification of foods marketed in Brazil according to the Brazilian and Mexican models

**DOI:** 10.3389/fnut.2022.919582

**Published:** 2022-09-20

**Authors:** Luiza Andrade Tomaz, Crislei Gonçalves Pereira, Luiza Vargas Mascarenhas Braga, Sarah Morais Senna Prates, Alessandro Rangel Carolino Sales Silva, Ana Paula da Costa Soares, Natália Cristina de Faria, Lucilene Rezende Anastácio

**Affiliations:** Food Science Graduation Program, Faculty of Pharmacy, Universidade Federal de Minas Gerais, Belo Horizonte, Minas Gerais, Brazil

**Keywords:** food labeling, nutrient profile, front-of-pack nutrition, labeling policies, food legislation, sweetners

## Abstract

Nutrient profiling is the science of classifying or ranking foods according to their nutritional composition, for reasons related to disease prevention and health promotion. To be effective, policies such as front-of-pack nutrition labeling (FoPNL) must have an adequate nutritional profile model, since it will determine which products will be eligible to receive a FoPNL. This study aimed to determine the percentage of packaged food and drink products available in Brazil that would be subject to FoPNL under two different legislations: Brazilian and Mexican. This is a cross-sectional study in which we collected information on food products (photos of the ingredients list, the front label, the barcode, and the nutrition facts table) from one of the largest stores of a supermarket chain in the city of Belo Horizonte-MG, Brazil, from March to May 2021 (~6 months after the publication of the Brazilian legislation about FoPNL and a year and a half before the legislation came into force). The products were classified in relation to the BNPM (added sugars, saturated fats, and sodium) and the MNPM (energy, free sugars, saturated fats, trans fats, sodium, non-sugar sweeteners, and caffeine). A total of 3384 products were collected and, after applying the exclusion criteria, 3,335 products were evaluated. Of these, 2,901 would be eligible to receive FoPNL in Brazil and 2,914 would be eligible to receive FoPNL in Mexico. According to the BNPM, 56.7% (95% CI 54.9; 58.5%) of the products were “high in” critical nutrients, 27.1% (95% CI 25.5; 28.7%) of the products in added sugars, 26.7% (95% CI 25.2; 28.4%) of the products in saturated fats, and 21.4% (95% CI 19.9; 22.9%) of the products in sodium. As for the MNPM, 96.8% (95% CI 96.1; 97.4%) of them were “high in” up to five critical nutrients and up to two warning rectangles (caffeine and non-sugar sweeteners), 45.8% (95% CI 44.0; 47.6%) of them in free sugars, 43.7% (95% CI 41.9; 45.5%) of them in saturated fats, and 47.9% (95% CI 46.1; 49.7%) of them in sodium. We concluded that the eligibility to receive FoPNL by BNPM and MNPM was relatively similar between products; however, almost all products would have at least one FoPNL and/or warning rectangles according to Mexican legislation, and nearly half of them would have at least one FoPNL, considering BNPM. The MNPM is much more restrictive than the BNPM. The Nutrient Profile Model (NPM) that regulates FoPNL, and other health policies, must be carefully defined to ensure that foods are properly classified according to their healthiness.

## Introduction

Food labeling is considered an important tool for promoting healthy eating habits, allowing consumers to have access to information on the nutritional composition of foods and thus conscious choices (1, 2). However, such information is difficult to understand and limits the potential of labeling as an effective method of communication of the nutritional content of foods (1, 3).

Due to these difficulties and as a strategy to promote healthier diets, following recommendations by the World Health Organization (WHO) (4, 5), several countries have already adopted front-of-pack nutrition labeling (FoPNL) on food packages (6–9). This type of labeling consists of simple and quick information about the nutritional quality of foods and is displayed on the main panel of labels to complement the nutritional information detailed on the back of packages and facilitate consumers' understanding of the composition of the products (5, 10). Evidence suggests that FoPNL facilitates the interpretation of information by consumers and favors healthier choices and purchases, in addition to contributing to the reformulation of food by the industry (11–19).

Focused on the main objective of better informing consumers about the composition of foods, different models of FoPNL have been implemented all over the world (2), and warning labels such as the octagon have been recently implemented in some countries in Latin America (6–9). In Brazil, the chosen model for the implementation of FoPNL was the black magnifying glass model, which will inform, from October 2022, the high content of added sugars, saturated fats, and sodium (20, 21). Mexico has adopted, since 2020, the FoPNL model in the shape of a black octagon, warning about the excess of calories; free sugars; saturated fats; trans fats and sodium and the presence of caffeine and non-sugar sweeteners [with the warning rectangle “*contiene cafe*í*na* (caffeine) *evitar en niños*” and “*contiene edulcorantes* (non-sugar sweeteners)—*no recomendable en niños*”] (6).

For the implementation of FoPNL, in addition to the label type and design, a Nutrient Profile Model (NPM) must also be defined (10). According to the WHO (4), nutrient profiling is the science of classifying or ranking foods according to their nutritional composition, for reasons related to disease prevention and health promotion. Such profiles use algorithms or cutoff points to convert the levels of nutrients and other food components into ratings or scores (22). The NPM also establishes eligibility criteria determining which foods will be able to be classified and will receive FoPNL and which nutrients will be considered, with their cutoff limits and the definition of food categories (23, 24). A careful definition of the NPM is essential to ensure that FoPNL helps consumers to differentiate less healthy foods from healthy foods and, consequently, to promote an improvement in the quality of diets (4, 25).

Currently, different NPMs are used around the world for different policy applications (4, 24, 25). The Pan American Health Organization (PAHO)'s NPM (26) was developed by experts in the field of nutrition, and it identifies processed and ultra-processed foods with excessive amounts of free sugars, sodium, total fats, saturated fats, and trans-fatty acids and informs about the presence of non-sugar sweeteners (26, 27). Ultra-processed foods are exclusive formulations of ingredients, resulting from a series of industrial processes (28). The PAHO's NPM was adapted in Mexico as the basis for defining the NPM of the current FoPNL regulation (Table 1) (6).

**Table 1:** Eligibility criteria and parameters of Brazilian and Mexican nutrient profile models.

	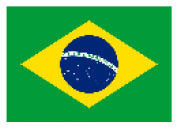 **Brazilian nutrient Profile** model **(BNPM) (IN 75, 2020)**	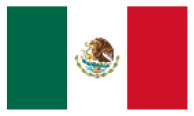 **Mexican nutrient** **Profile model (MNPM) (NOM-051, 2020)**			
FoPNL	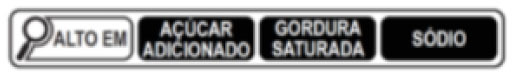	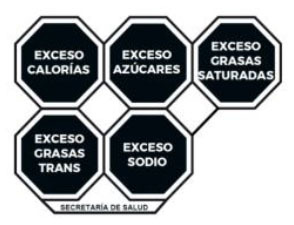			
FoPNL eligible products	Pre-packaged foods whose amounts of added sugars, saturated fats, or sodium are equal to or greater than the defined limits	Pre-packaged products with added free sugars, fats, or sodium and with the energy value, amount of free sugars, saturated fat, trans fat, and sodium equal to or greater than the defined limits			
FoPNL exempt products	✓ Fruits, vegetables, leguminous, tubers, cereals, nuts, chestnuts, seeds and mushrooms^*^ ✓ Flours^*^ ✓ Packaged, chilled, or frozen meat and fish^*^ ✓ Eggs^*^ ✓ Fermented milk^*^ ✓ Cheeses^*^ ✓ Milk of all species of mammalian animals ✓ Powdered milk ✓ Olive oil and other vegetable oils, cold-pressed or refined ✓ Salt for human consumption ✓ Infant formulas ✓ Enteral nutrition formulas ✓ Weight control foods ✓ Food supplements ✓ Alcoholic beverages ✓ Products intended exclusively for industrial processing or food service ✓ Food additives and technology adjuvants	✓ Infant formulas and follow-on formula ✓ Non-alcoholic foods and beverages for infants and young children with nutritional specifications for fats, sugars, and sodium ✓ Vegetable oils, vegetable or animal fats, sugar, honey, iodized salt, and fluoridated iodized salt, as well as cereal flours			
	**Solids/100 g**	**Liquids/100 mL**	**Solids/100 g**		**Liquids/100 mL**
Sugars	≥15 g Added sugar	≥7.5g Added sugar^a^	≥10% of total energy from free sugars^b^		
Saturated fats	≥6 g	≥3 g	≥10% of total energy from saturated fats		
Sodium	≥600 mg	≥300 mg	≥1 mg of sodium per kcal or ≥300 mg **Calorie-free drinks:** ≥45 mg of sodium		
Energy	NA	NA	≥275 total kcal		≥70 total kcal or ≥8 kcal from free sugars
Trans fats	NA	NA	≥1% of total energy from trans fats		
Non-sugar sweeteners	NA	NA	Presence		
Caffeine	NA	NA	Presence		

* As long as no ingredients that increase the added sugars value or significant nutritional value of saturated fats or sodium are added to the product, according to the established limits.

NA, not applicable (nutrient/ingredient not considered).

a Added sugar considering Brazilian Legislation are all monosaccharides and disaccharides added during food processing, including fractions of monosaccharides and disaccharides from the addition of the ingredients such as cane sugar, beet sugar, sugars from other sources, honey, molasses, “rapadura,” cane juice, extract malt, sucrose, glucose, fructose, lactose, dextrose, inverted sugar, syrups, maltodextrins, and other hydrolyzed carbohydrates and ingredients with the addition of any of the foregoing ingredients, with the exception of polyols, added sugars consumed by fermentation or non-enzymatic browning and sugars naturally present in milk and dairy products and sugars naturally present in vegetables, including fruits (whole, in pieces, in powder, dehydrated, in pulps, in purees, in whole juices, in reconstituted juices, and in concentrated juices) (21). In the present study, we could not consider maltodextrins as added sugar in the estimation of added sugars.

b Free sugars, considering Mexican Legislation, are available monosaccharides and disaccharides added (or added sugars) to foods and non-alcoholic beverages by the manufacturer, in addition to sugars that are naturally present in honey, syrups, and fruit or vegetable juices (6).

In Brazil, the NPM considered for the application of FoPNL (for added sugars, saturated fats, and sodium) was developed by the National Health Surveillance Agency (ANVISA) (Table 1). Before the publication of the new legislation on food nutrition labeling (20, 21), it was presented in a public consultation (20, 21, 29) that the NPM would be implemented in a staggered way to provide time for the food industry to adapt to these new labeling rules. However, in the new Brazilian legislation (20, 21), only the most flexible profile was considered. An estimate of the eligible products “high in” critical nutrients in Brazil was previously carried out, but either with stricter criteria and not officially implemented (27) or with a limited number of products (10). Thus, it is unknown, so far, what percentage of food and drink products in a Brazilian market would be eligible to receive FoPNL at the time that precedes the implementation of FoPNL in Brazil. Moreover, the Brazilian Nutrient Profile Model (BNPM) is more flexible than the current NPM adopted in countries that had already implemented FoPNL, such as Chile (30), Peru (9), and Uruguay (7). As the NPM is the first step to other public health policies, such as FoPNL, a more recent evaluation (6 months after the publication of the new Brazilian Legislation) in a large dataset of products available in the Brazilian food supply would be interesting to evaluate the performance of BNPM and compare it to a more restrictive model, like the MNPM.

Considering that the established criteria in the NPM are fundamental for the success and credibility of FoPNL, and other health policies that are dependent on NPM, this study aimed to evaluate and compare (for the common critical nutrient between the profiles) eligible food and drink products that would receive FoPNL according to the parameters of the BNPM and the MNPM.

## Materials and methods

### Study design

This was a cross-sectional study, in which packaged foods and drinks sold in Brazil were evaluated using the nutrition facts table, list of ingredients, and nutritional claims and classified according to the criteria of the BNPM and the MNPM. The comparison between the two NPMs was performed based on the respective eligibility and exclusion criteria for applying the FoPNL and the nutrients/substances, as well as their respective cutoff points according to the legislation of both countries (6, 20, 21).

### Data collection

Labeling information was collected at a supermarket in the city of Belo Horizonte-MG, between March and May 2021, by previously trained collectors, in one of the 10 largest chains in Brazil in 2020, and with prior authorization. The choice of the supermarket was based on the ranking published by the Brazilian Association of Supermarkets (ABRAS—Associação Brasileira de Supermercados). Data were collected from all foods and drinks that had a nutrition facts table according to the current Brazilian Regulation (RDC 360/2003) (31) and were available for sale during the collection period. If a product was available in multiple sizes or flavors, all flavors and all sizes would be collected. The products were categorized according to Normative Instruction n°75/2020 (20), a Brazilian regulation that divides foods into eight food groups: Group I–Bakery products, cereals, leguminous, roots, tubers, and their derivatives; Group II–Vegetables, greens, and pickled vegetables; Group III–Fruits, juices, nectars, and fruit refreshments; Group IV–Milk and dairy products; Group V–Meat and eggs; Group VI–Oils, fats, and oilseeds; Group VII–Sugars and products with energy from carbohydrates and fats; and Group VIII–Sauces, ready-to-eat seasonings, broths, soups, ready-to-eat dishes, and alcoholic beverages. The categories that make up the food groups are described in Supplementary Table 1.

Epicollect5 software (https://five.epicollect.net/), a free mobile and web application that generates questionnaires and freely hosts project websites for data collection, was used. The following information was collected from the packaging of the products: commercial name, sales denomination, flavor, net content, brand, barcode, nutritional information (energy and nutrients of concern), and ingredients list (added caffeine and non-sugar sweeteners). Concerning sugar content, this information was collected when it was available since the declaration of sugars is voluntary according to the Brazilian Legislation in force during data collection (31). For products without sugar content information but with sugars and/or foods that contain sugars in their ingredients list, an estimate of the content of free and added sugars was performed using an adapted method described by Scapin et al. (32) and the Pan American Health Organization (PAHO) (26).

To verify which products would receive the information “contains caffeine,” according to MNPM, the terms “coffee” and “cola” were searched in the sales denomination of the products, and among the selected products, the presence of added caffeine was searched in the list of ingredients. According to current Brazilian legislation, RDC 259/2002 (33), if caffeine is an ingredient in the product, it must be included in its ingredient list. For the evaluation of the presence of non-sugar sweeteners, Resolution RDC 18/2008 (34), a regulation of non-sugar sweeteners in Brazil, was consulted. Based on this document, a search was made for the following terms in the ingredients list: sorbitol, sorbitol syrup, D-sorbite, mannitol, acesulfame potassium, aspartame, cyclamic acid and its calcium, potassium and sodium salts, isomalt, isomaltitol, saccharin and its calcium, potassium, and sodium salts, sucralose, thaumatin, steviol glycosides, neotame, maltitol, maltitol syrup, lactitol, xylitol, erythritol, and advantame. Variations in listed names were considered, such as “stevia.”

### Application of eligibility and NPM thresholds

The BNPM and the MNPM were applied to eligible food and drink products according to described criteria in Table 1. Foods and drinks are eligible for the application of the NPM according to BNPM if they are added by ingredients that add significant nutritional value to the product, referring to sugars, saturated fat, and sodium above certain values (21). For the MNPM, for foods with added sugar, fat, or sodium and foods with energy, free sugars, saturated fat, trans fat, and sodium, as well as for foods containing non-sugar sweeteners and added caffeine, the above reference values are the target of FoPNL (6). The criteria adopted for the nutrient cut-off point were according to stage three of MNPM.

It is important to note that the MNPM also considers free sugars present in foods, considering sugars that are naturally present in honey, syrups, and fruit or vegetable juices, besides added sugars (6). The BNPM considers only added sugars. According to the BNPM, all monosaccharides and disaccharides are added during food processing including fractions of monosaccharides and disaccharides from the addition of the ingredients such as cane sugar, beet sugar, sugars from other sources, honey, molasses, “rapadura,” cane juice, extract malt, sucrose, glucose, fructose, lactose, dextrose, inverted sugar, syrups, maltodextrins, and other hydrolyzed carbohydrates and ingredients with the addition of any of the foregoing ingredients, with the exception of polyols, added sugars consumed by fermentation or non-enzymatic browning and sugars naturally present in milk and dairy products, and sugars naturally present in vegetables, including fruits (whole, in pieces, in powder, dehydrated, in pulps, in purees, in whole juices, in reconstituted juices, and in concentrated juices) (21). Although they are polysaccharides, maltodextrins were considered in the definition of added sugar of BNPM. In the present study, added sugar was estimated without considering the maltodextrins of BNPM. We assumed that the added sugar was equal to free sugar most of the time, except in the case of fruit juice addition (considered in the case of beverages).

According to MNPM, for products intended to be reconstituted or that require preparation before consumption, the declaration must be made following the directions for use indicated on the label. Therefore, chocolate powder, puddings, flans, ice cream powder, and cake mixes were calculated following its instructions (6). On the cotrary, BNPM, when referring to FoPNL, does not consider the nutritional value of the added ingredients to apply the NPM (20, 21).

**Figure 1:**
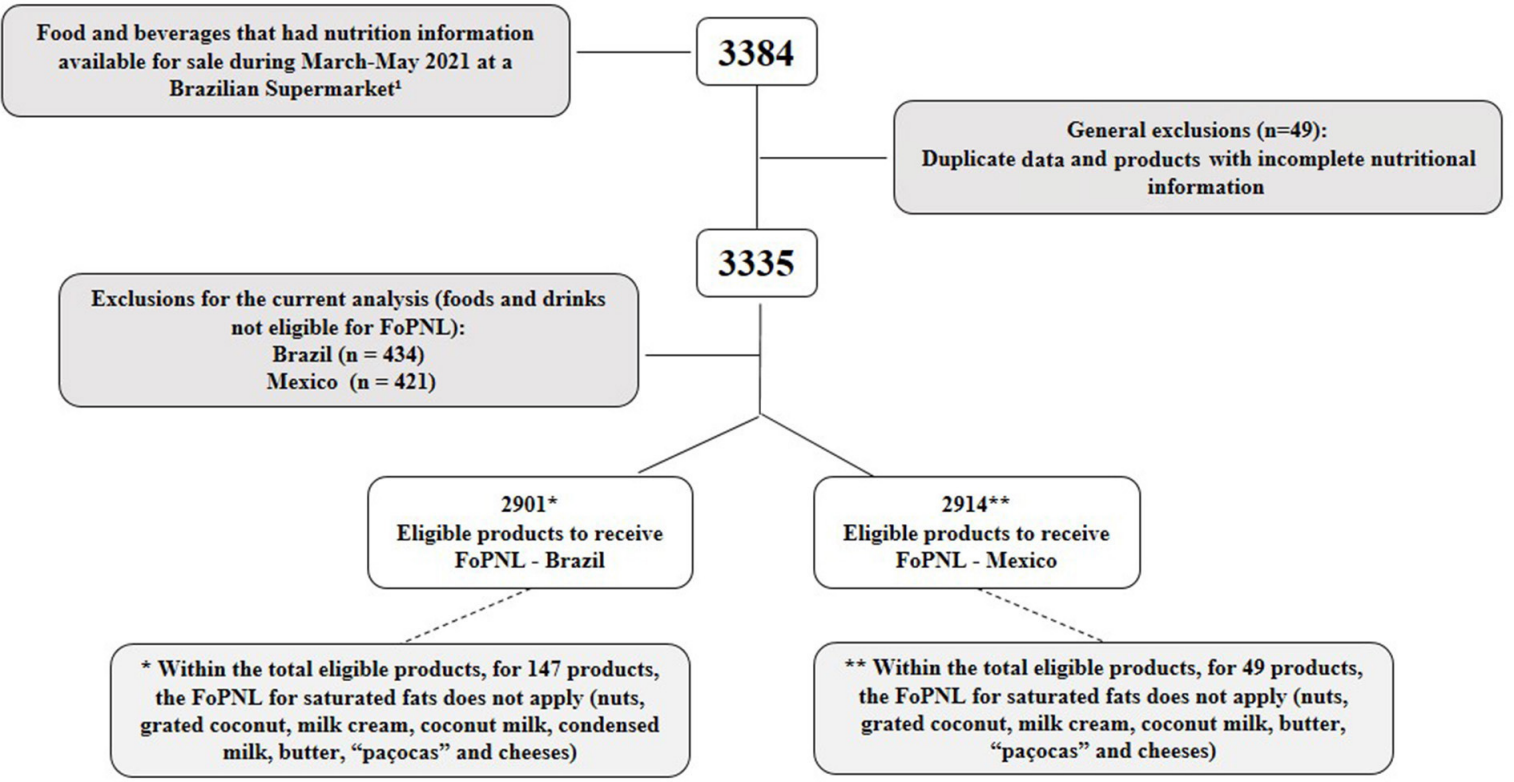
Number of initial and final products that were collected and used in the present study. ^1^Information table is not currently required for alcoholic beverages, food additives, and technology adjuvants the spices; natural mineral waters ans other waters for human consumption; to vinegars; to salt (sodium chloride); coffee, yebra mate, tea, and other herbs without the addition of other ingredients; food prepared and packed in restaurants and establishments commercial, ready for consumption; Fractionated products at retail points of sale, marketed as pre-measured; fresh, chilled, and frozen fruits, vegetables, and meats; food with packaging whose visible surface for labeling is ≤100cm^2^.

After applying the eligibility criteria, the foods whose nutritional labeling was not applicable were removed, and a total of 2,901 eligible products for FoPNL according to BNPM and 2,914 eligible products for FoPNL according to MNPM were obtained. It is worth mentioning that, within the total number of products eligible for FoPNL, in which saturated fat is intrinsic to its composition, the parameters for FoPNL of this nutrient were not applied for 147 foods for BNPM and 49 for MNPM (Figure 1).

### Statistical analysis

Data were compiled in an Excel spreadsheet (Microsoft Office). SPSS software (Statistical Package for Social Sciences) version 20.0 was used in the analyses. Numerical variables were presented as mean and standard deviation and also as median and interquartile range, given the non-normal distribution of the data (Kolmogorov–Smirnov test). The results of the eligibility and presence of FoPNL in the different food groups according to BNPM and MNPM were expressed in proportions and the 95% confidence interval was estimated using the binomial distribution as a reference. To compare the food and drink products “high in” values of sugars, saturated fats, and sodium, according to the BNPM and the MNPM, we used the McNemar test. To compare the values of sugars, saturated fat, and sodium among “high in” food and drink products according to the BNPM and the MNPM, we used the Mann–Whitney test. The adopted significance level was 5%.

## Results

The largest number of evaluated products belonged to Groups VII (*n* = 1269) and I (*n* = 679). It is noteworthy that 87.4% (95% CI 86.2; 88.5%) of the total products would be eligible for FoPNL considering MNPM and 87.0% (95% CI 85.8; 88.1%) considering BNPM. In Groups III and VII, the number of eligible products is close to the total (99.4%; 95% CI 97.3; 100% and 98.0%; 95% CI 97.2; 98.7%, respectively), and in Groups I and VI, the number of eligible products is the lowest observed (BNPM: 65.4% 95% CI 61.8; 68.9% and 65.1% 95% CI 58.7; 71.3%, respectively, and MNPM: 65.5% 95% CI 61.9; 69.0% and 66.1% 95% CI 59.6; 72.1%, respectively) (Table 2). The results considering the sub-groups of each group are described in Supplementary Table 1.

**Table 2:** Total number of collected food and drinks products, eligible for front-of-pack nutrition labeling (FoPNL), and receiving it according to Mexican and Brazilian Nutrient Profile Models.

**Food groups**			**Group I**	**Group II**	**Group III**	**Group IV**	**Group V**	**Group VI**	**Group VII**	**Group VIII**
	**Total**	**3,335^1^**	**679**	**84**	**161**	**505**	**135**	**218**	**1,269**	**284**
Eligible	Brazil	87.0 [85.8; 88.1]	65.4 [61.8; 68.9]	97.6 [92.8; 99.6]	99.4 [97.3; 100.0]	90.3 [87.5; 92.7]	96.3 [92.2; 98.7]	65.1 [58.7; 71.3]	98.0 [97.2; 98.7]	85.6 [81.2; 89.3]
	Mexico	87.4 [86.2; 88.5]	65.5 [61.9; 69.0]	97.6 [92.8; 99.6]	99.4 [97.3; 100.0]	90.3 [87.5; 92.7]	96.3 [92.2; 98.7]	66.1 [59.6; 72.1]	98.0 [97.2; 98.7]	89.1 [85.1; 92.4]
Presence FoPNL	Brazil	56.7 [54.9; 58.5]	40.5 [36.0; 45.2]	43.9 [33.5; 54.7]	1.9 [0.5; 3.8]	40.8 [36.3; 45.3]	60.0 [51.4; 68.2]	64.1 [56.0; 71.7]	70.6 [68.0; 73.1]	79.8 [74.5; 84.6]
	Mexico	96.8 [96.1; 97.4]	98.0 [96.4; 99.0]	95.1 [89.0; 98.5]	66.2 [58.7; 73.3]	99.1 [98.0; 99.7]	97.7 [94.1; 99.4]	100 [98.7; 100.0]	98.8 [98.1; 99.3]	98.4 [96.6; 99.5]
Added Sugars	Brazil	27.1 [25.5; 28.7]	9.2 [6.8; 12.2]	0.0 [0.0; 0.0]	1.2 [0.2; 3.8]	11.2 [8.5; 14.3]	0.0 [0.0; 0.0]	4.9 [2.1; 9.3]	54.3 [51.5; 57.0	4.12.9 [2.1; 7.1]
Free sugars	Mexico	45.8^*^ [44.0; 47.6]	22.9^*^ [19.2; 27.0; 28.6]	28.0 [19.1; 38.3]	53.8^*^ [46.0; 61.4]	50.2^*^ [45.6; 54.8]	0 [0.0; 0.0]	5.6 [2.6; 10.1]	68.6^*^ [66.0; 71.2]	13.0^*^ [9.3; 17.6]
Saturated fats	Brazil	26.7 [25.2; 28.4]	13.1 [10.1; 16.4]	3.7 [0.9; 9.2]	0.6 [0.0; 2.7]	27.9 [23.9; 32.1]	37.7 [29.7; 46.2]	16.2 [10.8; 22.8]	37.8 [35.1; 40.5]	18.5 [14.0; 23.7]
	Mexico	43.7^*^ [41.9; 45.5]	31.0^*^ [26.8; 35.4]	3.7 [0.9; 9.2]	1.2 [0.2; 3.8]	42.8^*^ [38.3; 47.3]	81.5^*^ [74.3; 87.6]	71.5^*^ [63.8; 78.5]	52.5^*^ [49.7; 55.3]	28.5^*^ [23.1; 34.2]
Sodium	Brazil	21.4 [19.9; 22.9]	25.7 [21.8; 29.9]	43.9 [33.5; 54.7]	0.0 [0.0; 0.0]	11.0 [8.3; 14.0]	55.4 [46.8; 63.8]	48.6 [40.5; 56.8]	7.2 [5.9; 8.8]	78.2 [72.7; 83.1]
	Mexico	47.9^*^ [46.1; 49.7]	75.1^*^ [70.9; 78.9]	90.2^*^ [82.6; 95.4]	5.0 [2.3; 9.1]	45.6^*^ [41.1; 50.2]	94.6^*^ [89.8; 97.6]	63.2^*^ [55.1; 70.8]	26.1^*^ [23.7; 28.6]	92.1^*^ [88.3; 95.0]
Calories	Mexico	72.9 [71.3; 74.5]	74.4 [70.2; 78.3]	29.3 [20.2; 39.7]	53.8 [46.0; 61.4]	76.3 [72.3; 80.1]	28.5 [21.2; 36.6]	78.5 [71.3; 84.6]	87.1 [85.1; 88.8]	40.3 [34.4; 46.4]
Trans fat	Mexico	5.0 [4.2; 5.8]	8.3 [6.0; 11.1]	0.0 [0.0; 0.0]	0.6 [0.0; 2.7]	8.3 [6.0; 11.1]	4.6 [1.9; 9.1]	9.7 [5.6; 15.3]	3.2 [2.3; 4.3]	3.6 [1.7; 6.3]
Non-sugar sweeteners	Mexico	15.8 [14.5; 17.2]	10.8 [8.1; 13.9]	12.2 [6.3; 20.4]	21.2 [15.4; 28.0]	13.8 [10.9; 17.2]	0.0 [0.0; 0.0]	4.2 [1.7; 8.3]	23.7 [21.4; 26.1]	2.0 [0.7; 4.2]
Caffeine	Mexico	0.6 [0.4; 0.9]	0.0 [0.0; 0.0]	0.0 [0.0; 0.0]	0.0 [0.0; 0.0]	0.0 [0.0; 0.0]	0.0 [0.0; 0.0]	0.0 [0.0]	1.4 [0.9; 2.2]	0.0 [0.0; 0.0]

1 The number of 3335 refers to the total products analyzed, however the percentages from the presence of FoPNL are based on the number of eligible products (BNPM=2901 BNPM and MNPM=2914).

Group I, Bakery products, cereals, leguminous, roots, tubers, and their derivatives; Group II, Vegetables, greens, and pickled vegetables; Group III, Fruits, juices, nectars, and fruit refreshments; Group IV, Milk and dairy products; Group V, Meat and eggs; Group VI, Oils, fats, and oilseeds; Group VII, Sugars and products with energy from carbohydrates and fats; Group VIII, Sauces, ready-to-eat seasonings, broths, soups, ready-to-eat dishes, and alcoholic beverages.

* p < 0.01 McNemar test for comparison of products “high in” according to Mexican and Brazilian Nutrient Profile Models for sugars, saturated fat, and sodium.

Of the 2,901 evaluated products according to the BNPM, 1255 (43.3%) products were not “high in” any critical nutrient, 1110 (38.3%) were “high in” one critical nutrient, 535 (18.4%) were “high in” two critical nutrients, and only 1 (0.03%) product was “high in” three critical nutrients. The product “high in” three critical nutrients comes from the category of sweet cookies, with or without filling (360 kcal/100 g; 43.3 g of added sugars/100 g; 7.2 g of saturated fat/100 g; 958.3 mg of sodium/100 g). The most prevalent critical nutrient that exceeded the threshold of the BNPM was saturated fat, present in 776 products (26.7% 95% CI 25.2; 28.4%), followed by added sugar, present in 786 products (27.1%; 95% CI 25.5; 28.7%), and sodium, present in 621 products (21.4% 95% CI 19.9; 22.9%) (Table 2). As for the MNPM, of the 2,914 evaluated products, 93 (3.2%) would receive no FoPNL, 441 (15.1%) would receive one FoPNL, 1,065 (36.5%) would receive two FoPNL, 1,090 (37.4%) products would receive three FoPNL, 216 (7.4%) products would receive four FoPNL, 9 (0.3%) products would receive five FoPNL, and no product would receive six or seven FoPNL. For both models, some food categories would have 100% of the products “high in” at least one critical nutrient (Supplementary Table 1).

The percentages of products high in sugars, saturated fats, and sodium were high in the MNPM compared to the BNPM (Sugars: 45.8%; 95% CI 44.0; 47.6 vs. 27.1%; 95% CI 25.5; 28.7% | Saturated fats: 43.7%; 95% CI 41.9; 45.5 vs. 26.7%; 95% CI 25.2; 28.4% | Sodium: 47.9%; 95% CI 46.1; 49.7 vs. 21.4%; 95% CI 19.9; 22.9%) (Table 2). We highlight the discrepancy of products “high in” sugars between the two legislations for Group III (53.8%; 95% CI 46.0; 61.4% of products by the MNPM against 1.2%; 95% CI 0.2; 3.8% of products by the BNPM). It was also observed that 25.7% (95% CI 21.8; 29.9%) of products in Group I and 43.9% (95% CI 33.5; 54.7%) of products in Group II were “high in” sodium by the BNPM *vs*. 75.1% (95% CI 70.9; 78.9%) (Group I) and 90.2% (95% CI 82.6; 95.4%) (Group II) of products by the MNPM (Table 2). For products classified as “high in” sugar, the group with higher prevalence considering the BNPM was Group VII (54.3%; 95% CI 50.5; 57.0%). Considering MNPM, Group VII is the group with the highest percentage of products “high in” free sugars (68.6%; 95% CI 66.0; 71.2%), followed by Group III (53.8%; 95% CI 46.0; 61.5%) and Group IV (50.2%; 95% CI 45.6; 54.8%). The last two groups include juices and nectars and fruit refreshments (Group III) and milk and dairy products (Group IV). The prevalence of “high in” added sugars in these groups considering the BNPM was only 1.2% (95% CI 0.2; 3.8%) and 11.2% (95% CI 8.5; 14.3%), respectively.

Regarding non-sugar sweeteners, these were found in 15.8% (95% CI 14.5; 17.2%) of the 2,914 products eligible for MNPM. Considering all the evaluated products (*n* = 3,335), this percentage is 13.8%. Group VII was the group with a higher percentage of non-sugar sweeteners, in which 23.7% (95% CI 21.4; 26.1%) of the products had additives. In addition, it was found that, in some sub-groups of food categories, 100% of the products contained non-sugar sweeteners: cakes of all types, without filling (*n* = 14), powders to prepare flans and desserts (*n* = 13), and vegetables, fruits, and soy juices (*n* = 5) (Supplementary Table 1). For caffeine, the presence was less than 1% in general (0.6%; 95% CI 0.4; 0.9%) and 1.4% (95% CI 0.9; 2.2%) in all products from Group VII (Table 2), with the non-alcoholic non-carbonated beverages, such as tea and soft drinks with the highest percentage (1.6%, 18 items of 112 items) (Supplementary Table 1).

The amounts of the targeted nutrients by BNPM were higher in products “high in” considering BNPM than the amounts of these same nutrients in products “high in” the MNPM. Products “high in” the BNPM have 24.0% higher levels of saturated fat (considering mean values: 12.9 vs. 10.4 g); 45.6% higher levels of sugars (28.1 vs. 19.3 g); and 98.8% higher levels of sodium (3,832.7 vs. 1,928.4 mg) in relation to the average values of products “high in” by the MNPM (Table 3).

**Table 3:** Mean and standard deviation, median, and interquartile intervale of nutrient contents for the food group with the presence and absence of front-of-pack nutrition labeling (FoPNL).

**FoPNL**	**Mexico (*****n*** = **2,914)**		**FoPNL**	**Brazil (*****n*** = **2,901)**		***p*** **value**
	**PRESENCE**	**ABSENCE**		**PRESENCE**	**ABSENCE**	
**Sugars**			**Sugars**			
Percentage (number of products)^a^	45.8% (1,340)	44.2% (1,574)	Percentage (number of products)^a^	27.1% (786)	72.9% (2,115)	< 0.001
Mean (standard deviation)	19.3 g (±16.7)	0.6 g (±1.6)	Mean (standard deviation)	28.1 g (±16.6)	1.9 g (±3.5)	
Median (IQR)^b^	14.3 g (6.6–28.0 g)	0 g (0–0 g)	Median (IQR)^b^	21.0 g (16.5–40.0 g)	0 g (0.0–2.8 g)	< 0.001
**Saturated fats**			**Saturated fats**			
Percentage (number of products)^a^	43.7% (1,272)	56.3% (1,642)	Percentage (Number of products)^a^	26.7% (776)	73.3% (2,125)	<0.001
Mean (standard deviation)	10.4 g (±8.7)	0.8 g (±1.4)	Mean (standard deviation)	12.9 g (±6.9)	1.2 g (±6.9)	
Median (IQR)^b^	8.0 g (5.0–14.4 g)	0 g (0–1 g)	Median (IQR)^b^	11.3 g (8.0–16.7 g)	0 g (0.0–2.3 g)	< 0.001
**Sodium**			**Sodium**			
Percenta g e (number of products)^a^	47.9% (1,396)	52.1% (1,518)	Percentage (Number of products)^a^	21.4% (621)	78.6% (2,280)	<0.001
Mean (standard deviation)	1,928.4 mg (±4,114.7)	38.6 mg (±38.4 mg)	Mean (standard deviation)	3,832.7 mg (±4,132.6)	166.4 mg (±4,108.9)	
Median (IQR)^b^	531.0 mg (340.0–856.0 mg)	52.5 mg (10.8–128.5 mg)	Median (IQR)^b^	970.0 mg (684.1–1,809.1 mg)	86.7 mg (31.9–276.5 mg)	<0.001

IQR, Interquartile Range.

a Mc-Nemar test;

b Mann–Whitney Test.

## Discussion

This study compared the application of Mexican and Brazilian NPM on 3384 collected products in the Brazilian market, 6 months after the publication and one and a half years before Resolution n° 429 of 2020 came into force (21). Considering the MNPM, only 3.2% of the products would not receive FoPNL, while by the BNPM, almost half of the products (43.3%) would be classified as healthy. These results corroborate with the results of other studies that compared the BNPM with the PAHO's NPM and/or MNPM and found higher percentages of foods classified as healthy for the first profile (27, 35). Duran et al. (27) evaluated a preliminary and less rigorous BNPM than the one approved by the Brazilian Legislation, used in the present study, and observed that 38% and 55% of the foods were classified as healthy (without FoPNL) by the PAHO's NPM and BNPM, respectively. In the study conducted by Contreras-Manzano et al. (35), who evaluated foods available in the Mexican market, about 20% and almost half of the products were classified as healthy by the MNPM and the BNPM, respectively. Despite the methodological differences between the aforementioned and the present study, it is possible to notice an overestimation of the percentage of products classified as healthy by the BNPM.

Less strict NPMs, which fit a greater number of products with lower nutritional quality into healthy eating standards (36), are less capable of improving consumer eating behaviors (37) and cannot encourage product reformulation by the industry, maintaining the levels of harmful nutrients to health (38). In addition, NPMs can be used for various other purposes related to the prevention and control of obesity and overweight (26, 39) and also in addition to FoPNL, such as regulating the use of nutrition and health claims on foods, regulating the marketing of unhealthy foods to children, taxes on unhealthy foods, and restrictions on foods and beverages available or sold in and out of schools (40–42).

Although the eligibility criteria are different between the MNPM and the BNPM, especially regarding products that are exempted from FoPNL, there were no significant differences in the proportion of eligible foods for both NPMs. This result can be justified because, although the BNPM exempts a larger variety of foods from receiving FoPNL compared to the MNPM, most of these products become eligible for FoPNL when added to ingredients containing sugars, saturated fats, and sodium, bringing these results closer. This demonstrates that many of the products sold in a Brazilian supermarket and displaying the nutrition facts tables are ultra-processed or processed, aligned with other surveys of foods by the degree of processing and by NPM in supermarkets in Brazil (43) and other countries (44, 45). Eligibility criteria are important to protect some food categories that should be the main source of human food and nutrition—such as unprocessed or minimally processed foods, from the NPM and the consequent health policies, such as FoPNL. Also, applying eligibility criteria before the thresholds of NPM is important to predict a scenario assessment closer to reality.

The higher number of products “high in” sugars, saturated fat, and sodium by the MNPM compared to the BNPM is consistent with results from other studies comparing the PAHO's NPM with other NPMs (46–50), in which PAHO's is stricter and classifies a greater proportion of foods as “unhealthy.” This result can be justified by the stricter cutoff points in the MNPM compared to the BNPM. Also, free sugars (considered in the MNPM and that includes the sugar of fruits and vegetable juices to the added sugars) are different from added sugars (considered in BNPM). The huge differences in the prevalence of products “high in” sugars in Group III (Fruits, juices, nectars, and fruit refreshments) are a consequence of the different definitions adopted between countries, besides the different cutoffs.

Some specific criteria for the application of FoPNL are also plausible justifications for the higher proportion of foods identified as “high in” by the MNPM. For example, in products that require preparation before consumption, the MNPM considers both the nutrients in the food itself and the nutrients in the added ingredients (6). On the contrary, BNPM, despite considering reconstitution, since the limits for the application of FoPNL are considered based on the ready-to-eat food, only considers the nutrients of the food itself, without the nutritional value of the added ingredients (20).

In the present study, only five products that would receive FoPNL according to the BNPM for saturated fat would not be “high in” for the same nutrient according to the MNPM: a 50% soluble cocoa chocolate powder, a corn snack, and three wheat snacks would receive FoPNL for saturated fat. The high energy density of the products is one possible explanation for this. If a food, not only has a high content of a certain nutrient, in this case, saturated fat, but also has a high energy content, the proportion is maintained and there is no extrapolation of the MNPM cutoff point, since, for these nutrients, the measure is relative (10% of the energy value) and not absolute. Although the number of products in this situation is small, this observation has been previously reported (10, 48). It is worth mentioning that the same products were “high in” for calories (all), one for free sugars (the 50% soluble cocoa chocolate powder), and the snacks for sodium according to the MNPM. Acording to MNPM, 5% of products would be “high in” trans fat.

In Brazil, trans fat was not considered in the NPM, since the legislation published in 2019 in the country foresees the limitation of the use of this component in foods (51). According to that regulation, partially hydrogenated fat will be banned as of 1 January 2023 (51).

The prevalence of caffeine in products was only 0.6% in the present study. Contreras-Manzano et al. (35) recently evaluated 38,872 packaged food products available in the Mexican supermarket and found a prevalence of 0.8% of products with added caffeine. We are not aware of other studies that identified the prevalence of added caffeine in food, probably due to the design of food labeling regulations, which make it difficult to identify them in food. We cannot discard the possibility of sub-estimation of added caffeine prevalence considering the way it has been researched in our products. However, the importance of including this information more clearly on labels has already been raised (52) since studies point to possible health harms through caffeine consumption, such as convulsions (53, 54), liver and kidney damage (55), cardiac arrhythmias (56), and headache (57). Furthermore, in children, caffeine consumption is associated with impaired growth and development, which justifies the inclusion of the warning retangle in MNPM (58).

The present study indicated that 13.8% of all evaluated products contained non-sugar sweeteners in their composition (15.8% considering eligibility criteria). Previous studies with data from 2013 (13.3%) (59) and 2017 (9.3%) (23) also reported the prevalence of non-sugar sweeteners in Brazil. However, considering the presence of non-sugar sweeteners in food categories, it was observed that some of them have different values from those previously found. While in this study, 100% of powders for preparing flans and desserts (*n* = 13) had at least one non-sugar sweetener, in the study by Figueiredo et al. (59), and the prevalence was 58.3% (*n* = 24). In addition, of the 63 evaluated soft drinks in this study, 71.4% contain one or more non-sugar sweeteners (data not shown), while in the study by Grilo et al. (23), of the 106 evaluated soft drinks, 44.3% had the additive. It should be noted that the higher prevalence of the use of non-sugar sweeteners in the group of soft drinks may be a consequence of the rules of the new Brazilian regulation of nutrition labeling (20, 21), since all soft drinks that do not have the addition of non-sugar sweeteners and were evaluated in this study would be “high in” added sugars by BNPM. Thus, the adoption of an NPM that has a warning rectangle for non-sugar sweeteners could avoid the reformulation of foods with the replacement of free sugars with non-sugar sweeteners. In Chile, whose NPM does not foresee the adoption of a warning for the presence of non-sugar sweeteners, the use of the additive increased from 37.9 to 43.6% after the initial implementation of the Chilean Labeling Law (60). It is important to notice that the use of other non-sugar sweeteners has emerged in countries that adopted the NPM—such as monk fruit and allulose—and the health effects of this kind of reformulation should be studied in future (35).

It is important to note that 45 of the 461 evaluated products with non-sugar sweeteners are products that have declared polyols in the ingredients list with a moistening, emulsifying, or stabilizing function. However, by the definition of the MNPM, non-sugar sweeteners are substances other than monosaccharides and disaccharides that impart a sweet flavor to products (6). Thus, even if they do not have the function of partially or completely replacing sugar, they are counted as products with non-sugar sweeteners and must carry the warning rectangle “*contiene edulcorantes (non-sugar sweeteners)—no recomendable en niños*.” We highlighted here the result found in the category “cakes, all types, without filling” (*n* = 14), where 100% of the products had a non-sugar sweetener substance but with another technological function described in its ingredients list.

For this study, to the best of our knowledge, data collection took place most recently in Brazil. Data collection happened between March and May 2021, when the changes provided by the new food labeling legislation, expected to be implemented in October of 2022, (20, 21), were already known. However, the present study has some limitations that deserve discussion. First, the collection was restricted to only one supermarket in the city of Belo Horizonte and may not reflect all the available packaged foods for sale in the country. Second, the assessment of the presence of FoPNL for sugars was performed based on estimates of free and added sugars (26, 32), since in Brazil, to date, the declaration of the total sugar content of foods is not mandatory. Also, BNPM considers maltodextrin as added sugar and it was not possible to estimate the amount of maltodextrin in some products. The research for added caffeine can be sub-estimated since we searched for caffeine in some products that, by sales denomination, probably had caffeine and not in all products of our database. Finally, the different ways of categorizing a database (22, 61) can make it difficult to compare the results from different studies, for example, in the categorization used in this study, soft drinks are included in the category of non-alcoholic beverages, carbonated, or non-carbonated (teas, soy-based drinks, and soft drinks). However, this was the categorization that best suited the database products and has also been used in other studies (59, 62).

## Conclusions

Under both BNPM and MNPM, most of the evaluated products in this study were “high in” nutrients that are harmful to health. Although the percentage of products eligible to receive FoPNL was very close between the two profiles (87.4% under the MNPM and 87.0% under the BNPM), the total number of products “high in” critical nutrients varied greatly (96.8% under the MNPM and 56.7% under the BNPM). In addition, the application of the MNPM criteria resulted in higher proportions of products identified with an excess of each nutrient (sugars, saturated fats, and sodium) specifically, because it encompasses more nutrients than the BNPM, such as calories, trans fat, non-sugar sweeteners, and caffeine, but also because of the more restrictive cutoff points.

## Data availability statement

The raw data supporting the conclusions of this article will be made available by the authors, without undue reservation.

## Author contributions

LA: study conception. ASi, LT, LB, ASo, and CP: data collection and transcription. LT, CP, and LB: data categorization. CP, LB, ASi, and LT: data on eligibility. LB, CP, ASi, and ASo: FoPNL application and tabulation. SP, LT, and ASo: discussion of data. SP, LT, CP, LB, and LA: writing. ASi: translation. NF: formatting and references. All authors: sugar estimation and final version of approval.

## Funding

SUPPORT: Conselho Nacional de Desenvolvimento Científico e Tecnológico-CNPq and Ministério da Saúde-MS (442990/2019-7) and Fundação de Amparo à Pesquisa do Estado de Minas Gerais-FAPEMIG (APQ-00341-21). Pro-Reitoria de Pesquisa da Universidade Federal de Minas Gerais.

## Conflict of interest

The authors declare that the research was conducted in the absence of any commercial or financial relationships that could be construed as a potential conflict of interest.

## Publisher's note

All claims expressed in this article are solely those of the authors and do not necessarily represent those of their affiliated organizations, or those of the publisher, the editors and the reviewers. Any product that may be evaluated in this article, or claim that may be made by its manufacturer, is not guaranteed or endorsed by the publisher.
