# Supplementary material for: From the most to the least flexible nutritional profile: Classification of foods marketed in Brazil according to the Brazilian and Mexican models
Source: Front Nutr. 2022 Sep 20;9:919582. doi: 10.3389/fnut.2022.919582 (PMC9531871; doi:10.3389/fnut.2022.919582)
Supplement: Supplementary file 1 [file Table_1.pdf]

Supplementary Material

SUPPLEMENTARY TABLE 1. PROPORTION OF FOODS THAT WOULD RECEIVE FoPNL ACCORDING TO THE CRITERIA OF THE BRAZILIAN AND MEXICAN LEGISLATIONS, GENERAL, BY FOOD GROUP AND CATEGORY, BRAZIL, 2021

	Brazil				Mexico							
**Food groups and categories**	**N***	**Added sugars (%)**	**Saturated fat (%)**	**Sodium (%)**	**N***	**Calories (%)**	**Free sugars (%)**	**Saturated fats (%)**	**Trans fats (%)**	**Sodium (%)**	**Caffeine (%)**	**Sweeteners (%)**
**Group I – Bakery products, cereals, leguminous, roots, tubers and their derivatives**	**444**	**9.2**	**13.1**	**25.7**	**445**	**77.1**	**22.9**	**31.0**	**8.3**	**75.1**	**0.0**	**10.8**
Starches	0	-	-	-	0	-	-	-	-	-	-	-
Raw rice	0	-	-	-	0	-	-	-	-	-	-	-
Oat flakes without other ingredients	0	-	-	-	0	-	-	-	-	-	-	-
Cereal bars with up to 10% of fat	15	40.0	0.0	6.7	15	100.0	60.0	20.0	0.0	6.7	0.0	20.0
Pre-fried frozen potatoes and cassava	16	0.0	0.0	6.3	16	0.0	0.0	50.0	0.0	31.3	0.0	0.0
Potatoes, cassava and other tubers, cooked in water and vacuum-packed	0	-	-	-	1	0.0	0.0	0,0	0.0	0.0	0.0	0.0
Salted cookies. Whole meal and grissini	78	0.0	0.0	28.2	78	100.0	3.8	48.7	5.1	97.4	0.0	0.0
Cakes all kinds without filling	14	57.1	0.0	28.6	14	100.0	71.4	78.6	0.0	14.3	0.0	100.0
Canjica (rawgrain)	0	-	-	-	0	-	-	-	-	-	-	-
Whole raw cereals	0	-	-	-	0	-	-	-	-	-	-	-
Morning cereal weighing up to 45 g per cup	37	64.9	8.1	0.0	37	100.0	73.0	8.1	2.7	45.9	0,0	16.2
Morning cereal weighing more than 45 g per cup	1	0.0	100.0	0.0	1	100.0	0.0	100.0	0.0	0.0	0.0	0.0
Cereal bran and wheat germ	0	-	-	-	0	-	-	-	-	-	-	-

Breadcrumbs	0	-	-	-	0	-	-	-	-	-	-	-
Milk Flour	18	16.7	0.0	0.0	18	100.0	16.7	0.0	0.0	0.0	0.0	0.0
Cereal and tuber flours – all kinds	0	-	-	-	0	-	-	-	-	-	-	-
Ready-to-eat “farofa”	8	0.0	0.0	62.5	8	100.0	0.0	0.0	0.0	100.0	0.0	0.0
Dried leguminous. All	0	-	-	-	0	-	-	-	-	-	-	-
Dried pasta	0	-	-	-	0	-	-	-	-	-	-	-
Pizza dough	3	0.0	0.0	33.3	3	100.0	0.0	0.0	33.3	100.0	0.0	0.0
Dough for savory pies	2	0.0	100.0	0.0	2	100.0	0.0	100.0	0.0	100.0	0.0	0.0
Fresh pasta with and with out fillings	11	0.0	0.0	36.4	11	36.4	0.0	0.0	9.1	90.9	0.0	0.0
Pastry and pancake mixes	5	0.0	0.0	100.0	5	100.0	0.0	0.0	0.0	100.0	0.0	0.0
Packaged bread, sliced or not, with or without filling	76	0.0	0.0	1.3	76	48.7	3.9	3.9	0.0	94.7	0.0	6.6
Potato bread. Cheese bread and other chilled and frozen with filling and dough for breads	24	0.0	16.7	37.5	24	41.7	0.0	70.8	58.3	87.5	0.0	0.0
Potato bread, cheese bread and other cooled and frozen bread without filling, “chipa paraguaia”	24	0.0	16.7	25.0	24	62.5	8.3	70.8	12.5	87.5	0.0	8.3
Sweet bread withoutfruit	9	0.0	0.0	0.0	9	88.9	22.2	0.0	0.0	88.9	0.0	11.1
Popcorn	17	0.0	64.7	76.5	17	100.0	0.0	76.5	5.9	82.4	0.0	0.0
Cake and pie making powders	43	0.0	0.0	2.3	43	97.7	83.7	18.6	27.9	69.8	0,0	9.3
Powders for flans and desserts	13	0.0	0.0	0,0	13	53.8	53.8	53.8	0.0	69.2	0,0	100.0
Soy-based preparations (such as “milanesa”, meatballs and hamburger)	8	0.0	62.5	37.5	8	12.5	0.0	75.0	0.0	100.0	0.0	0.0
Pre-friedor frozen cereal and tuber products	1	0.0	0.0	0.0	1	0.0	0.0	0.0	0.0	100.0	0.0	0.0
Toasts	21	0.0	0.0	38.1	21	100.0	0.0	4.8	0.0	100.0	0.0	0.0
“Quibe” wheat and texturized soy	0	-	-	-	0	-	-	-	-	-	-	-

protein												
**Group II – Vegetables, greens and pickled vegetables**	**82**	**0.0**	**3.7**	**43.9**	**82**	**35.4**	**28.0**	**3.7**	**0.0**	**90.2**	**0.0**	**12.2**
Triple vegetable concentrate (extract)	11	0.0	0.0	45.5	11	9.1	0.0	0.0	0.0	100.0	0.0	0.0
Vegetable and pickled vegetables (carrots. peas. corn. Peeled tomatoes and others)	7	0.0	0.0	0.0	7	0.0	0.0	0.0	0.0	85.7	0.0	0.0
Tomato sauce or sauce made from tomatoes and other vegetables	25	0.0	4,0	76.0	25	96.0	72.0	4.0	0.0	100.0	0.0	20.0
Vegetable purée or pulp. Including tomatoes	5	0.0	0.0	20.0	5	0.0	0.0	0.0	0.0	40.0	0.0	0.0
Juices from vegetables, fruits and soybeans	5	0.0	0.0	0.0	5	80.0	100.0	0.0	0.0	20.0	0.0	100.0
Dehydrated vegetables for soup	0	-	-	-	0	-	-	-	-	-	-	-
Pickled vegetables (artichoke, asparagus, mushrooms, bell peppers, cucumbers, and hearts of palm) in brine, vinegar and olive oil	29	0.0	6.9	37.9	29	0.0	0.0	6.9	0.0	100.0	0.0	0.0
**Group III – Fruits, juices, nectars and fruit refreshments**	**160**	**1.3**	**0.6**	**0.0**	**160**	**91.9**	**53.8**	**1.3**	**0.6**	**5.0**	**0.0**	**21.3**
Canned fruit. Including fruit salad	4	0.0	0.0	0.0	4	100.0	25.0	0.0	0.0	0.0	0.0	50,0
Dried fruit (pears, peaches, pineapples, plums. edible parts)	8	12.5	12.5	0.0	8	100.0	12.5	12.5	12.5	12.5	0.0	0.0
Fruitpulp for refreshment. Concentrated fruit juice and dehydrated fruit juice	30	0.0	0.0	0.0	30	63.3	43.3	0.0	0.0	6.7	0.0	0.0
Juice, nectar and fruit drinks	117	0.9	0.0	0,0	117	98.3	60.7	0.9	0.0	4.3	0.0	27.4
Raisins	1	0.0	0.0	0.0	1	100.0	0.0	0.0	0.0	0.0	0.0	0.0
**Group IV – Milk and dairy products**	**456**	**11.2**	**27.9**	**11.0**	**456**	**80.5**	**50.2**	**42.8**	**8.3**	**45.6**	**0.0**	**13.8**
Dairy drink	74	45.9	2.7	1.4	74	85.1	73.0	24.3	0.0	28.4	0.0	16.2

Powdered milk	0	-	-	-	0	-	-	-	-	-	-	-
Fluid milk. All kinds	0	-	-	-	0	-	-	-	-	-	-	-
Fermented milks, yogurts. All kinds	197	7.6	0.0	0.0	197	87.8	74.1	0.0	2.5	21.3	0.0	22.3
Other cheeses (ricotta. semi-hard. white. cream cheese. melted and paste)	127	0.0	96.1	33.1	127	68.5	0.0	97.6	24.4	92.9	0.0	0.0
Powders for preparing dairy desserts	0	0.0	0.0	0.0	0	0,0	0,0	0,0	0,0	0,0	0,0	0
Ice cream powders	7	0.0	0.0	0.0	7	100.0	100.0	100.0	0.0	0.0	0.0	0.0
Cottage cheese. Skimmed ricotta cheese. minas cheese. skimmed cream cheese and petit-suisse	32	0.0	3.1	0.0	32	59.4	46.9	90.6	3.1	53.1	0,0	9.4
Grated cheese	8	0.0	0.0	87.5	8	100.0	0.0	87.5	12.5	100.0	0.0	0.0
Dairy desserts	11	18.2	18.2	0.0	11	90.9	63.6	90.9	0.0	18.2	0.0	36.4
**Group V – Meat and eggs**	**130**	**0.0**	**37.7**	**55.4**	**130**	**28.5**	**0,0**	**81.5**	**4.6**	**94.6**	**0.0**	**0.0**
Meatballs based on meat	2	0.0	50.0	0.0	2	0.0	0.0	100.0	50.0	100.0	0.0	0.0
Tuna. sardines. seafood, shellfishes. Other canned fish with or without sauce	20	0.0	0.0	5.0	20	0.0	0.0	40.0	0.0	95.0	0.0	0.0
Sausages. ham	18	0.0	94.4	100.0	18	88.9	0.0	100.0	0.0	100.0	0.0	0.0
Meat-based hamburger	13	0.0	38.5	53.8	13	15.4	0.0	100.0	7.7	92.3	0.0	0.0
Kani-kama	1	0.0	0.0	0,0	1	0,0	0.0	0.0	0.0	0,0	0.0	0.0
Sausageall kindsds	34	0.0	55.9	88.2	34	41.2	0.0	94.1	0.0	100.0	0.0	0.0
Pâtés (ham. liver and bacon etc)	4	0.0	0.0	0.0	4	0.0	0.0	25.0	0.0	100.0	0.0	0.0
Meat preparations with flour or breaded	8	0.0	12.5	12.5	8	0.0	0.0	100.0	25.0	100.0	0.0	0.0
Preparations of seasoned. smoked. Cooked or uncooked meats	30	0.0	20.0	50.0	30	16.7	0.0	80.0	6.7	86.7	0.0	0.0
**Group VI – Oils, fats and oilseeds**	**142**	**4.9**	**16.2**	**48.6**	**144**	**80.6**	**5.6**	**71.5**	**9.7**	**63.2**	**0.0**	**4.2**
**Olives**	23	0.0	8.7	100,0	23	4.3	0.0	82.6	0.0	100.0	0.0	0.0

Bacon pieces – smoked or fresh	2	0.0	0,0	100,0	2	50,0	0.0	0.0	0.0	100.0	0.0	0.0
Lard and animal fats	1	0.0	0,0	100,0	1	100.0	0.0	0.0	0.0	100.0	0.0	0.0
Whipping cream	3	0.0	33.3	0,0	3	100.0	33.3	66.7	0.0	0,0	0.0	33.3
Shredded coconut	14	50.0	0,0	0,0	14	100.0	50.0	57.1	0.0	0,0	0.0	0.0
Milk cream	17	0.0	0,0	0,0	17	100.0	0.0	0.0	5.9	17.6	0.0	0.0
Coconut milk	0	-	-	-	0	-	-	-	-	-	-	-
Mayonnaise and mayonnaise-based sauces	16	0.0	25,0	87.5	16	68.8	0.0	93.8	0.0	100.0	0.0	0.0
Butter, margarine and similar	48	0.0	6.3	56.3	48	100.0	0.0	87.5	25.0	79.2	0.0	0.0
Vegetable oils. All kinds	0	-	-	-	0	-	-	-	-	-	-	-
Oilseeds (mixed. cut. chopped. whole)	18	0.0	72.2	11.1	20	100.0	0.0	85.0	5.0	40.0	0.0	25.0
**Group VII – Sugars and products with energy from carbohydrates and fats**	**1244**	**54.3**	**37.8**	**7.2**	**1244**	**90.4**	**68.6**	**52.5**	**3.2**	**26.1**	**1.4**	**23.7**
Chocolate similar powder. cocoa-based powders. chocolate powder and cocoa powder	48	64.6	2.1	4.2	48	97.9	77.1	77.1	6.3	35.4	0.0	18.8
Sugar. All kinds	0	-	-	-	0	-	-	-	-	-	-	-
Candies. lollipops and chewing gum	57	94.7	14.0	0.0	57	96.5	94.7	15.8	0.0	1.8	0.0	12.3
Cereal bars with more than 10% fat. torrones. “pé de moleque” and “paçoca”	24	20.8	8.3	0.0	24	100.0	45.8	16.7	0.0	0.0	0.0	25.0
Non-alcoholic beverages. Carbonated or noncarbonated (teas. soy-based drinks and soft drinks)	112	32.1	0.0	0.0	112	65.2	59.8	3.6	0.0	0.0	16.1	54.5
Sweet cookie. with or without filling	285	79.3	57.9	1.4	285	100.0	85.6	70.5	3.9	19.6	0.0	14.0
Cakes and similar products with filling or topping	25	92.0	36.0	0.0	25	100.0	88.0	68,0	0.0	0.0	0.0	76.0

Brownies and alfajores	2	100.0	0.0	0.0	2	100.0	100.0	0.0	0.0	0.0	0.0	50.0
Chocolates. trufles and similar	152	87.5	96.1	0.7	152	100.0	88.2	96.7	0.0	2.0	0.0	11.2
Chocolate confectionery and dragees in general	20	85.0	50.0	0.0	20	100.0	90.0	90.0	30.0	0.0	0.0	0.0
Stickysweetcandies (guava. quince. fig. potato. etc)	28	60.7	14.3	0.0	28	100.0	85.7	32.1	3.6	7.1	0.0	10.7
Sweets in paste (pumpkin. guava. milk. banana. mocotó)	22	27.3	40.9	0.0	22	100.0	59.1	86.4	0,0	4.5	0.0	36.4
Candiedfruit	1	100.0	0.0	0.0	1	100.0	100.0	0.0	0.0	0.0	0.0	0.0
Jams	43	46.5	0.0	0.0	43	90.7	62.8	0.0	0.0	0.0	0.0	30.2
Corn glucose. honey. molasses. fruittoppings. condensed milk and other syrups (cassis. currant. raspberry. blackberry. guaraná etc)	15	100.0	0.0	0.0	15	100.0	100.0	53.3	0.0	0.0	0.0	0.0
Chewing gum	8	12.5	0.0	0.0	8	12.5	12.5	0.0	0.0	0.0	0.0	87.5
Mix for the preparation of sweets, icing for cakes, pies and ice cream etc.	2	100.0	0.0	0.0	2	100.0	100.0	50.0	0.0	0.0	0.0	0.0
Jelly powder	55	1.8	0.0	23.6	55	56.4	56.4	0,0	0,0	94.5	0,0	94.5
Powders for the preparation of refreshment	66	22.7	0.0	0.0	66	47.0	80.3	0.0	0.0	5.0	0.0	68.2
Snacks based on cereals and snack flours	175	0.6	53.7	40.0	175	96.0	0.6	53.7	4.6	90.9	0.0	0.0
Ready-madejelly dessert	1	100.0	0.0	0.0	1	100.0	100.0	0.0	0.0	0.0	0.0	0.0
Ice creams	99	64.6	22.2	0.0	99	99.0	92.9	82.8	11.1	0.0	0.0	7.1
Individual ice creams	4	100.0	0.0	0.0	4	100.0	100.0	75.0	0.0	25.0	0.0	0.0
**Group VIII – Sauces, ready-to-eat seasonings, broths, soups, ready-to-eat dishes and alcoholic beverages**	**243**	**4.1**	**18.5**	**78.2**	**253**	**41.1**	**13.0**	**28.5**	**3.6**	**92.1**	**0.0**	**2.0**

Broth (meat. chicken. vegetables etc.) and souppowdersincluding (bori-bori. pirá caldo. shoyu)	34	0.0	38.2	61.8	34	38.2	0.0	41.2	0.0	100.0	0.0	0.0
Ketchup and mustard	25	24.0	0.0	92.0d	26	84.6	61.5	0.0	0.0	92.3	0.0	19.2
Dairy-based sauces or gravies	4	0.0	50.0	25.0	4	75.0	25.0	75.0	0.0	100.0	0.0	0.0
Soy or vinegar-based sauces	52	7.7	9.6	94.2	52	48.1	28.8	17.3	0.0	98.1	0.0	0.0
Ready and semi-ready prepared dishes not included in other items in the table	61	0.0	41.0	54.1	68	50.0	0.0	67.6	13.2	80.9	0.0	0.0
Complete seasoning	67	0.0	0.0	94.0	69	10.1	1.4	0.0	0.0	94.2	0.0	0.0
**Total**	2901	27.1	26.7	21.4	2914	77.7	45.8	43.6	5.0	47.9	0.6	15.8
